# Based on Network Pharmacology Tools to Investigate the Molecular Mechanism of Cordyceps sinensis on the Treatment of Diabetic Nephropathy

**DOI:** 10.1155/2021/8891093

**Published:** 2021-02-05

**Authors:** Yan Li, Lei Wang, Bojun Xu, Liangbin Zhao, Li Li, Keyang Xu, Anqi Tang, Shasha Zhou, Lu Song, Xiao Zhang, Huakui Zhan

**Affiliations:** ^1^Hospital of Chengdu University of Traditional Chinese Medicine, Chengdu, 610072 Sichuan, China; ^2^Key Laboratory of Chinese Internal Medicine of Ministry of Education and Dongzhimen Hospital, Beijing University of Chinese Medicine, Beijing 100700, China; ^3^Zhejiang Chinese Medical University, Hangzhou, 310053 Zhejiang, China

## Abstract

**Background:**

Diabetic nephropathy (DN) is one of the most common complications of diabetes mellitus and is a major cause of end-stage kidney disease. Cordyceps sinensis (Cordyceps, Dong Chong Xia Cao) is a widely applied ingredient for treating patients with DN in China, while the molecular mechanisms remain unclear. This study is aimed at revealing the therapeutic mechanisms of Cordyceps in DN by undertaking a network pharmacology analysis.

**Materials and Methods:**

In this study, active ingredients and associated target proteins of Cordyceps sinensis were obtained via Traditional Chinese Medicine Systems Pharmacology Database (TCMSP) and Swiss Target Prediction platform, then reconfirmed by using PubChem databases. The collection of DN-related target genes was based on DisGeNET and GeneCards databases. A DN-Cordyceps common target interaction network was carried out via the STRING database, and the results were integrated and visualized by utilizing Cytoscape software. Gene Ontology (GO) and Kyoto Encyclopedia of Genes and Genomes (KEGG) pathway enrichment analyses were performed to determine the molecular mechanisms and therapeutic effects of Cordyceps on the treatment of DN.

**Results:**

Seven active ingredients were screened from Cordyceps, 293 putative target genes were identified, and 85 overlapping targets matched with DN were considered potential therapeutic targets, such as TNF, MAPK1, EGFR, ACE, and CASP3. The results of GO and KEGG analyses revealed that hub targets mainly participated in the AGE-RAGE signaling pathway in diabetic complications, TNF signaling pathway, PI3K-Akt signaling pathway, and IL-17 signaling pathway. These targets were correlated with inflammatory response, apoptosis, oxidative stress, insulin resistance, and other biological processes.

**Conclusions:**

Our study showed that Cordyceps is characterized as multicomponent, multitarget, and multichannel. Cordyceps may play a crucial role in the treatment of DN by targeting TNF, MAPK1, EGFR, ACE, and CASP3 signaling and involved in the inflammatory response, apoptosis, oxidative stress, and insulin resistance.

## 1. Introduction

Diabetic nephropathy (DN) is featured as hyperglycemia, hyperfiltration, proteinuria, and progressive renal function decline, which can cause end-stage kidney disease [1]. It accounts for about 40% of chronic kidney disease worldwide and is undoubtedly a medical challenge worldwide [2], in terms of high incidence, multifactorial pathogenesis, and the absence of practical methods in the diagnosis and treatment [3]. Traditional Chinese medicine (TCM) guided by the unique theory provides an effective treatment of complex chronic diseases via multicomponent, multitarget, and multipathway [4]. In recent years, Chinese herb medicine has been commonly utilized to alleviate and reverse diabetes and its complications, such as DN in clinical practice and scientific researches, which has been considered a beneficial supplement of the drug therapy for DN [4]. Cordyceps, a traditional Chinese herbal medicine, is reported to have multiple health-promoting characteristics, including anti-inflammatory, anticancer, antidiabetic, analgesic, antioxidant, antiallergic, and antiobesity [5]. Also, Cordyceps has been reported to have broad pharmacological effects on DN by inhibiting the epithelial-mesenchymal transition, alleviating oxidative stress, repressing inflammation, modulating gut microbiota dysbiosis, and activating autophagy [6–9]. However, the pharmacological mechanisms of Cordyceps associated with DN only focus on a single chemical molecule. Accordingly, a comprehensive and systematic evaluation of the molecular mechanisms of Cordyceps on DN is indispensable.

**Figure 1:**
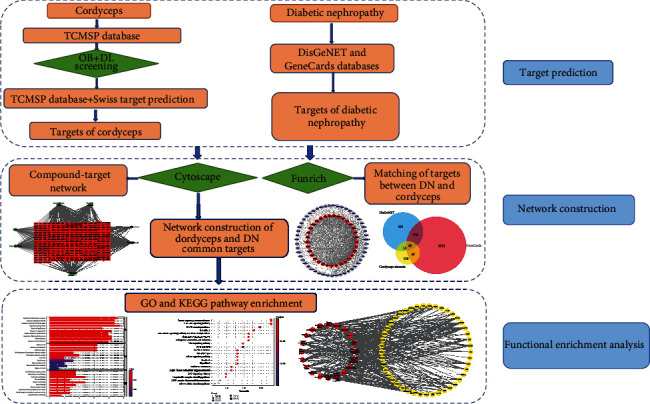
A flow chart based on an integration strategy of network pharmacology.

This study is aimed at analyzing the pharmacological mechanisms of active ingredients of Cordyceps involved in the progression of DN via using the network pharmacology databases and biological analysis methods. It laid a stable foundation for further research on exploring pharmacological mechanisms of Cordyceps in treating DN. The study framework is showed in Figure 1.

## 2. Materials and Methods

### 2.1. Establishment of Active Ingredients and Correlated Target Database

TCMSP (http://lsp.nwu.edu.cn/tcmsp.php), a systematic pharmacological platform that contains the relationships among herbal compounds, related targets, and diseases [10], was applied to identify the chemical constituents of Cordyceps. The active components were selected based on oral bioavailability (OB) and drug likeness (DL) values, and the ingredients were captured when the OB was ≥30% and the DL ≥ 0.18 (a screening threshold of TCMSP database) [10]. The chemical formulas of components were reconfirmed by PubChem (https://www.ncbi.nlm.nih.gov/pccompound) to double-check the final compounds of Cordyceps [11]. The targets associated with Cordyceps components were further identified based on the TCMSP database and Swiss Target Prediction (http://www.swisstargetprediction.ch.), a webserver to accurately identify the targets of active molecules [12]. The genes corresponding to the protein targets were obtained from the UniProt database.

### 2.2. Network Construction of Active Components-Potential Targets

**Figure 2:**
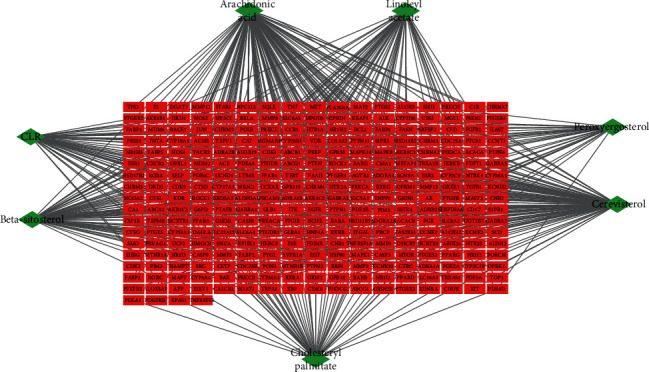
Compound-target network of putative targets and active Cordyceps ingredients. The green nodes represent the active components of Cordyceps, and nodes in red represent the corresponding targets of the ingredients.

A comprehensive network was constructed via using Cytoscape software to reflect the intricate relationship between active compounds and putative targets [13]. Nodes represent the components and targets, while edges reveal the interactions between components and targets (Figure 2).

### 2.3. Selection of Potential DN Targets

The keyword “diabetic nephropathy” was inputted in the GeneCards (https://www.genecards.org/), a human gene compendium with information about genomics, proteomics, and transcriptomics [14], and DisGeNET (https://www.disgenet.org/home/), a comprehensive platform including one of the largest publicly accessible collections of genes, to search for DN-associated targets [15].

### 2.4. Screening Compound-Disease Overlapping Targets

**Figure 3:**
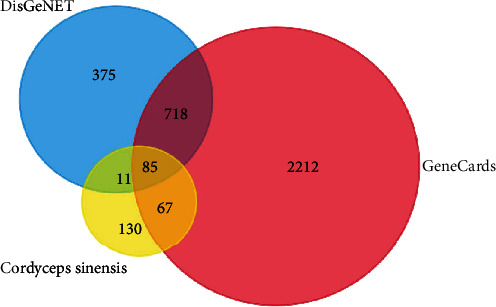
Overlapping target genes between DN and Cordyceps.

The screened compound targets and disease targets were imported into Funrich, a software used mainly for functional enrichment and interaction network analysis of genes and proteins for analysis [16]. The common targets of compound-disease were obtained as the potential targets for further analysis (Figure 3).

### 2.5. Network Construction of Compound-Disease Common Targets

A protein-protein interaction (PPI) network was obtained based on the STRING platform (https://string-db.org/), which covers nearly all functional interactions among the expressed proteins [17]. Target interaction information derived from the STRING database was imported into the Cytoscape (version 3.7.1; https://www.cytoscape.org/) software where the interaction information was integrated and analyzed.

### 2.6. GO and KEGG Pathway Enrichment Analyses

GO is the most comprehensive and widely used knowledgebase for the classification of gene functions, including the biological process (BP), molecular function (MF), and cell component (CC) [18]. KEGG (http://www.kegg.jp/) is an encyclopedia of genes and genomes; connecting genomic information to higher-order functional information to capture significantly enriched biological pathways is the major objective of the KEGG knowledgebase [19].

In our study, GO functional annotation and KEGG pathway enrichment analysis were carried out via using the ClusterProfiler package of R software, and *P* < 0.05 was employed as a screening threshold.

## 3. Results

### 3.1. Active Ingredients of Cordyceps

The active chemical components of Cordyceps were selected via the TCMSP databases, and 38 compounds were collected with the thresholds of OB ≥ 30% and DL ≥ 0.18 properties. Finally, 7 candidate ingredients were screened out for further study (Table 1).

### 3.2. Compound-Target Network Construction

In order to visualize the interaction relationship between the Cordyceps ingredients and corresponding targets, we established a compound-target network (Figure 2). By mapping 7 components to 293 potential targets, the network comprises 300 nodes and 500 edges, in which the red circles correspond to the putative targets and Cordyceps ingredients are in green. Chemical compound arachidonic acid, cerevisterol, beta-sitosterol, linoleyl acetate, cholesteryl palmitate, CLR, and peroxyergosterol correspond to 133, 100, 77, 57, 53, 49, and 31 targets, respectively. The results suggest that these 7 components probably serve significant therapeutic roles in DN.

### 3.3. Predicting DN-Related Targets

By retrieving the DisGeNET and GeneCards databases, results were integrated to obtain the DN-associated targets. As shown in Figure 3, the potential target genes in Cordyceps were matched to the DN-associated target gene by using the Funrich platform and was shown as a Venn diagram. Finally, 85 putative targets were selected according to the intersection of component targets and DN-related targets; one target was excluded because it had no interaction with other targets (Table 2).

### 3.4. Common Target Network

**Figure 4:**
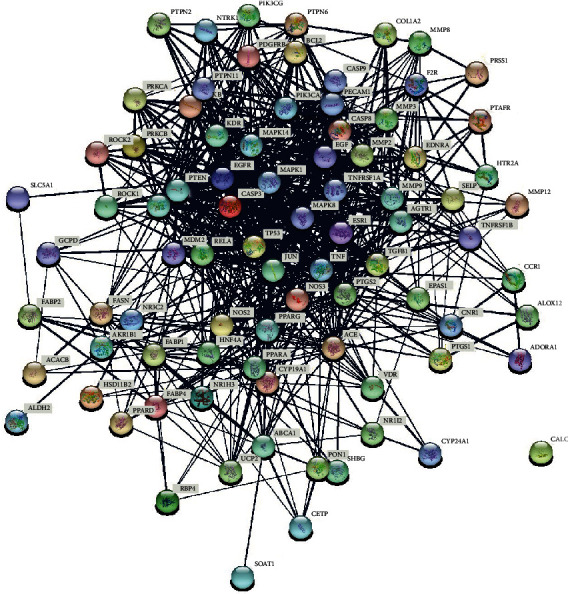
PPI network of overlapping targets between DN and Cordyceps. Each circular node represents a protein target, and the 3D structure in the circular nodes shows the protein spatial structure. The lines among different nodes represent the association among potential protein targets, while the width of lines was according to the action intensity.

**Figure 5:**
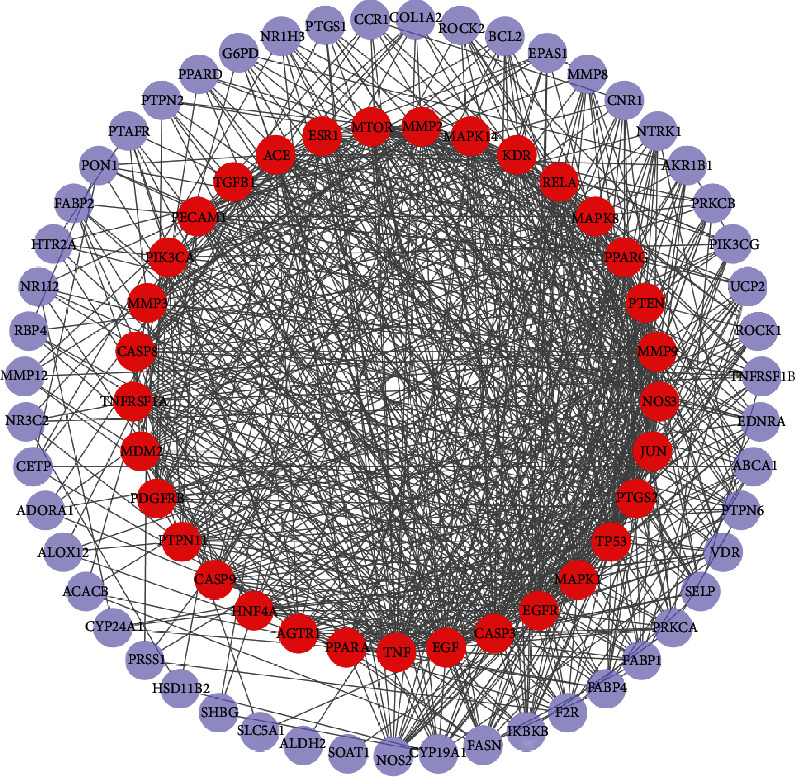
PPI network integration and analysis. The red nodes display the most potential targets in treating DN, which are greater than the average degree. Also, the nodes in purple show the potential targets under the average degree. Furthermore, the density of lines among inner nodes indicates the interaction relationship between different protein targets.

85 putative genes correlated with DN were imported to the STRING database for analysis and network establishment. The interaction network was based on the selected targets with a medium confidence score of 0.400 (Figure 4). A total of 84 nodes and 767 edges were embodied, and the average node degree is 18.3 after analysis. The results were imported to the Cytoscape software for further analysis and network construction (Figure 5). In this figure, the edges represent the interaction between a pair of potential targets, while the nodes represent the targets, and the degree value indicates the intensity of target interaction.

### 3.5. GO and KEGG Pathway Enrichment Analyses

**Figure 6:**
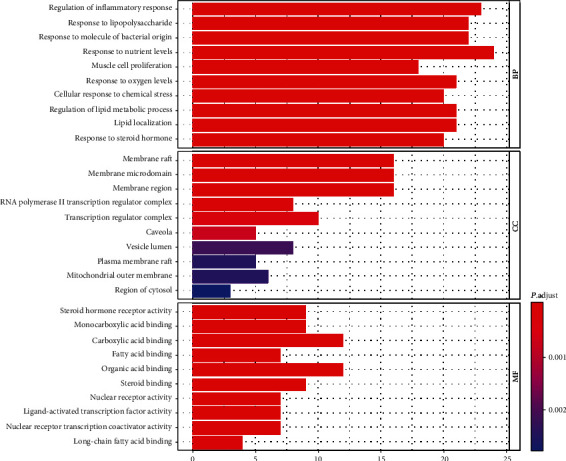
Enriched GO terms for BP, MF, and CC of putative targets of active components of Cordyceps. The color of the bar is displayed in a gradient from red to blue according to the ascending order of the *P* value, while the length of the bar is arranged according to the ascending order of the number of gene counts.

After using the ClusterProfiler package for pathway analysis, a total of 1843 biological processes were enriched. The top 10 remarkably enriched BP terms were selected for analysis, including regulation of inflammatory response, response to lipopolysaccharide, response to molecule of bacterial origin, response to nutrient levels, muscle cell proliferation, response to oxygen levels, cellular response to chemical stress, regulation of lipid metabolic process, and lipid localization. Besides, 32 cell components were enriched, and the top 10 entries were screened, consisting of membrane raft, membrane microdomain, membrane region, RNA polymerase II transcription regulator complex, transcription regulator complex, caveola, vesicle lumen, plasma membrane raft, mitochondrial outer membrane, and region of cytosol. Furthermore, a total of 118 molecular functions were enriched; the top 10 entries were selected, containing steroid hormone receptor activity, monocarboxylic acid-binding, carboxylic acid-binding, fatty acid-binding, organic acid-binding, steroid binding, nuclear receptor activity, ligand-activated transcription factor activity, nuclear receptor transcription coactivator activity, and long-chain fatty acid-binding. These processes are of great significance to further understand the curative mechanism of Cordyceps on the treatment of DN. The results of the GO analysis are illustrated in Figure 6.

**Figure 7:**
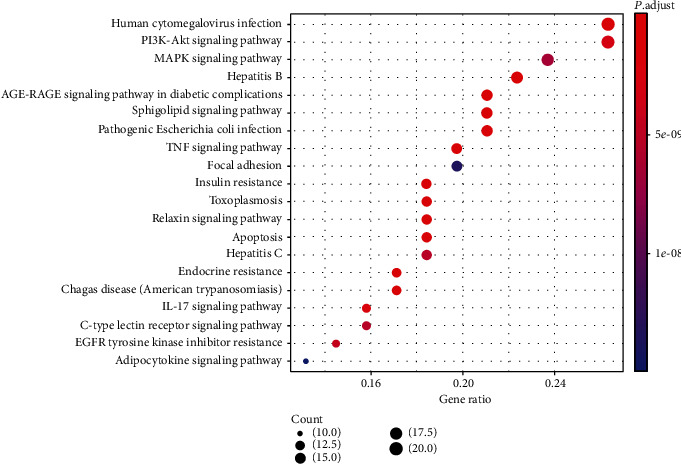
KEGG pathway analysis of potential targets of Cordyceps. The top 20 pathways are selected following the criteria of *P* < 0.05. The longitudinal axis represents the name of different pathways, and the transverse axis shows the number of enriched genes. Besides, the dot size represents the proportion of the number of enriched genes to the total number of genes, which is according to the descending proportion value. And the color of the dot is displayed in a gradient from red to blue according to the ascending order of the *P* value.

In terms of KEGG analysis, a total of 118 pathways were obtained. The top 20 significantly enriched pathways were screened out based on the threshold of *P* < 0.05 (Figure 7). The results indicated that these genes were mainly associated with the inflammatory signaling pathway, apoptosis, oxidative stress, and insulin signaling pathway, including AGE-RAGE signaling pathway in diabetic complications, TNF signaling pathway, apoptosis, IL-17 signaling pathway, PI3K-Akt signaling pathway, and insulin resistance. The results prove that Cordyceps may alleviate DN by regulating insulin resistance, apoptosis, and inflammatory reaction.

**Figure 8:**
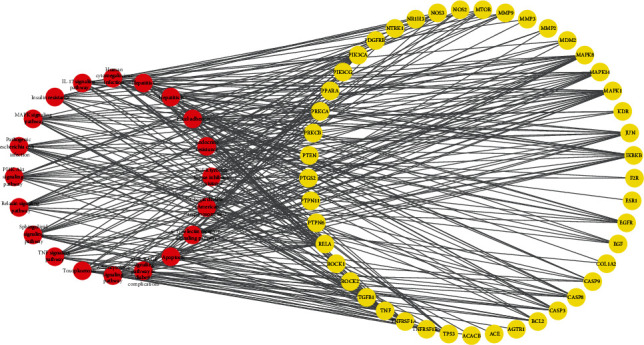
Pathway-target interaction network. Red circles indicate pathways which have interactions with potential targets, while the yellow circles represent putative targets.

To more intuitively demonstrate which pathway each target is involved in, the target pathway data captured from KEGG analysis was uploaded into Cytoscape software for constructing a network graph of target and pathway (Figure 8).

## 4. Discussion

DN, featured as high incidence, multifactorial pathogenesis, and absence of practical methods for diagnosis and treatment, is undoubtedly a medical challenge worldwide. The etiology of DN is multifactorial; with hyperglycemia, oxidative stress, and advanced glycation end products (AGE) as the leading factors, chronic inflammation and infiltrated immune cells in renal tissue are considered to be the common pathological consequences [20, 21]. Despite current improving therapies, there is still a considerable residual risk of DN onset and progression [22]. Some relevant studies highlight new perspectives of TCM for delaying DN progression and strengthen the therapeutic rationale for TCM on the treatment of DN [23, 24]. Cordyceps contains several active ingredients which affect multiple targets and pathways in the progression of DN and has been used as an adjuvant on the treatment of DN in China for a long time [7, 25]. In this study, we selected 7 active ingredients from Cordyceps based on the OB and DL, including linoleyl acetate, arachidonic acid, cholesteryl palmitate, CLR, beta-sitosterol, cerevisterol, and peroxyergosterol. Some compounds of Cordyceps were reported to have the effect on ameliorating endocrine and metabolic disorders during the development of DN [26–29]. For instance, it was reported that beta-sitosterol protects the expression of insulin signaling molecules through activating insulin receptor and glucose transporter 4 in the adipose tissue with a high-fat diet, which can slow the development of DN [29]. Arachidonic acid is a strong inducer of insulin secretion and it can attenuate DN by inhibiting the TGF-*β*/Smad signaling pathway [26, 30]. In addition, arachidonic acid also can facilitate the production of anti-inflammatory lipoxins which were reported to improve insulin sensitivity and may prevent the development of diabetes [27]. Besides, several arachidonic acid metabolites, including PGE2, PGI2, and LXA4, PGE2 and PGI2 can alleviate insulin resistance and improve insulin sensitivity of pancreatic cells [28]; LXA4 can inhibit the production of IL-6, TNF-*α*, and ROS, thus alleviating inflammation, and has an antidiabetic effect [31, 32].

We found that many targets can be regulated by multiple compounds from the compound-target network, such as CYP19A1, NOS2, NR1H3, SHBG, and PTGS2. When it comes to core targets, TNF (degree = 51), MAPK1 (degree = 51), EGFR (degree = 45), and CASP3 (degree = 45) played an important role in the process of Cordyceps in DN treatment. TNF and MAPK1 are correlated with inflammation response and deterioration of renal function [33, 34]. CASP3 is known to have an important role in the promotion of apoptotic cell death [35]. AG1478 can block EGFR signaling and inhibit oxidative stress and endoplasmic reticulum stress markers in diabetic mice [36]. In addition, inhibition of EGFR activation is associated with improved DN and insulin resistance in type 2 diabetes mouse models [37]. Therefore, the candidate targets are mainly enriched for oxidative stress, insulin resistance, apoptosis, and inflammation.

We also selected 85 common targets between the components of Cordyceps and DN for performing GO enrichment, consisting of biological processes, molecular function, and cellular components, which is aimed at predicting the mechanism of Cordyceps in treating DN. We found that the candidate targets are involved in multiple biological processes, such as regulation of inflammatory response, response to lipopolysaccharide, response to nutrient levels, response to oxygen levels, cellular response to chemical stress, regulation of lipid metabolic process, and lipid localization. The active targets such as TNF, PPARG, MAPK1, EGFR, and TGF-*β*1 mainly participate in the biological processes of inflammatory response, oxidative stress, and lipid metabolic process [36, 38, 39]. Thus, the molecular processes of several targets are relatively consistent with the pathogenesis and mechanism of clinical DN. In addition, cellular components constitute membrane raft, membrane microdomain, membrane region, RNA polymerase II transcription regulator complex, and transcription regulator complex. It indirectly illustrates the complexity of the pathogenesis of DN and its damage to several cellular components, and Cordyceps may have a function in regulating these cellular components, eventually improving DN. Besides, molecular functions are mainly enriched in steroid hormone-related activity and steroid-binding, and many studies reported that steroid hormones were closely related to DN in patients with diabetes [40, 41]. It reveals that Cordyceps might target steroid hormone in DN treatment.

Using network pharmacological analysis and performing KEGG enrichment, we found that these Cordyceps components may relieve the symptoms of DN through the action of targets in various signaling pathways and multiple biological processes, including AGE-RAGE signaling pathway, TNF signaling pathway, apoptosis, IL-17 signaling pathway, PI3K-Akt signaling pathway, and insulin resistance. Activation of the receptor for AGEs or RAGE (receptor for advanced glycation end-products) is associated with the development of DN [42], which evokes oxidative stress and chronic inflammation in renal tissues, ending up in losses in kidney function by activating various intracellular signalings like PI3K/Akt/mTOR, NF-*κ*B, MAPK/ERK, and TGF-*β*/Smad [43–45]. Additionally, it is believed that reactive oxygen species (ROS) can regulate PI3K/Akt/mTOR signaling and play an essential role in the development of DN, including epithelial-mesenchymal transition (EMT). During EMT, epithelial cells lose their primary epithelial properties, such as epithelial- (E-) cadherin, while acquiring characteristics typical of mesenchymal cells such as *α*-SMA, ending up with renal interstitial fibrosis [46]. Moreover, PI3K/Akt/mTOR signaling can promote high glucose-induced podocyte apoptosis, which contributes to the pathogenesis of DN [47].

TNF signaling is characterized as a well-known inflammatory cytokine associated with renal injury [48]. Once stimulated by TNF-*α*, NF-*κ*B moves from the cytoplasm to the nucleus and activates the transcription of VCAM-1, ICAM-1, IL-6, and IL-8, which will result in endothelial inflammatory and DN pathological process acceleration [49]. Furthermore, NF-*κ*B can be activated by several cytokines, which in turn induces the production of TNF-*α*, resulting in diabetic renal damage [50].

Insulin resistance is a critical process and one of the main symptoms in the initiation and progression of DN, which is closely related to microalbuminuria [51, 52]. High levels of insulin can cause insulin receptor degradation and drive early podocyte insulin resistance, and both the insulin receptor and nephrin are needed for full insulin sensitivity of podocytes. Thus, it explains why individuals with nephropathy caused by type 2 diabetes are commonly hyperinsulinaemic in the early stage of their disease [53]. Moreover, there are lots of mediators of insulin resistance that participate in driving renal function decline, including TGF-*β*1, blood pressure, inflammation, TNF-*α*, IL-6, and oxidative stress [54–56].

Therefore, these results of network pharmacological analyses not only verify that our screened targets are consistent with previous literature reports but also indicate that Cordyceps play a significant therapeutic role in DN by regulating several signaling pathways, including inflammatory response, insulin resistance, oxidative stress, apoptosis, and other pathways with unclear mechanisms. It will provide a novel methodology for further study of the therapeutic mechanism of Cordyceps in alleviating DN.

## 5. Conclusion

In summary, our network pharmacological analyses have shown that Cordyceps play an indispensable supplementary role in the treatment of DN, which is consistent with previous studies. Moreover, the biological functions of active chemical molecules and their corresponding targets of Cordyceps analyzed by network pharmacological methods provide a unique and innovative path for the study of TCM and further reveal the molecular biological mechanism of Cordyceps in treating DN. However, in vivo and in vitro experiments should be undertaken to validate the relationship between key targets and pathways of Cordyceps for the treatment of DN. Despite the limitations of this study, the results of this study provide new evidence and theoretical basis which will be used in subsequent theoretical and clinical research studies of Cordyceps.

## Figures and Tables

**Table 1:** Essential information about Cordyceps active components.

Mol ID	Molecule name	OB (%)	DL
MOL001645	Linoleyl acetate	42.1	0.2
MOL001439	Arachidonic acid	45.57	0.2
MOL008999	Cholesteryl palmitate	31.05	0.45
MOL000953	CLR	37.87	0.68
MOL000358	Beta-sitosterol	36.91	0.75
MOL008998	Cerevisterol	39.52	0.77
MOL011169	Peroxyergosterol	44.39	0.82

**Table 2:** Information of putative targets and topological attributes.

No.	UniProt ID	Protein names	Gene names	Degree
1	P01375	Tumor necrosis factor	TNF	51
2	P28482	Mitogen-activated protein kinase 1	MAPK1	45
3	P01133	Proepidermal growth factor	EGF	45
4	P00533	Epidermal growth factor receptor	EGFR	45
5	P42574	Caspase-3	CASP3	45
6	P04637	Cellular tumor antigen p53	TP53	44
7	P35354	Prostaglandin G/H synthase 2	PTGS2	41
8	P14780	Matrix metalloproteinase-9	MMP9	40
9	P29474	Nitric oxide synthase, endothelial	NOS3	40
10	P05412	Transcription factor AP-1	JUN	40
11	P37231	Peroxisome proliferator-activated receptor gamma	PPARG	37
12	P60484	Phosphatidylinositol 3,4,5-trisphosphate 3-phosphatase and dual-specificity protein phosphatase PTEN	PTEN	37
13	P45983	Mitogen-activated protein kinase 8	MAPK8	36
14	Q04206	Transcription factor p65	RELA	35
15	P35968	Vascular endothelial growth factor receptor 2	KDR	33
16	Q16539	Mitogen-activated protein kinase 14	MAPK14	33
17	P08253	72 kDa type IV collagenase	MMP2	32
18	P42345	Serine/threonine-protein kinase mTOR	MTOR	32
19	P03372	Estrogen receptor	ESR1	31
20	P12821	Angiotensin-converting enzyme	ACE	28
21	P01137	Transforming growth factor beta-1 proprotein	TGFB1	27
22	P16284	Platelet endothelial cell adhesion molecule	PECAM1	27
23	P42336	Phosphatidylinositol 4,5-bisphosphate 3-kinase catalytic subunit alpha isoform	PIK3CA	26
24	P08254	Stromelysin-1	MMP3	25
25	Q14790	Caspase-8	CASP8	25
26	P09619	Platelet-derived growth factor receptor beta	PDGFRB	23
27	Q00987	E3 ubiquitin-protein ligase Mdm2	MDM2	23
28	P19438	Tumor necrosis factor receptor superfamily member 1A	TNFRSF1A	23
29	P55211	Caspase-9	CASP9	22
30	Q06124	Tyrosine-protein phosphatase nonreceptor type 11	PTPN11	22
31	P41235	Hepatocyte nuclear factor 4-alpha	HNF4A	20
32	P30556	Type-1 angiotensin II receptor	AGTR1	20
33	Q07869	Peroxisome proliferator-activated receptor alpha	PPARA	20
34	P11511	Aromatase	CYP19A1	16
35	P35228	Nitric oxide synthase, inducible	NOS2	16
36	P49327	Fatty acid synthase	FASN	16
37	O14920	Inhibitor of nuclear factor kappa-B kinase subunit beta	IKBKB	16
38	P25116	Proteinase-activated receptor 1	F2R	15
39	P17252	Protein kinase C alpha type	PRKCA	15
40	P07148	Fatty acid-binding protein, liver	FABP1	15
41	P15090	Fatty acid-binding protein, adipocyte	FABP4	15
42	P16109	P-selectin	SELP	14
43	P11473	Vitamin D3 receptor	VDR	14
44	P29350	Tyrosine-protein phosphatase nonreceptor type 6	PTPN6	14
45	O95477	Phospholipid-transporting ATPase ABCA1	ABCA1	14
46	P25101	Endothelin-1 receptor	EDNRA	13
47	P55851	Mitochondrial uncoupling protein 2	UCP2	12
48	P48736	Phosphatidylinositol 4,5-bisphosphate 3-kinase catalytic subunit gamma isoform	PIK3CG	12
49	P05771	Protein kinase C beta type	PRKCB	12
50	Q13464	Rho-associated protein kinase 1	ROCK1	12
51	P20333	Tumor necrosis factor receptor superfamily member 1B	TNFRSF1B	12
52	P15121	Aldo-keto reductase family 1 member B1	AKR1B1	11
53	P21554	Cannabinoid receptor 1	CNR1	11
54	P04629	High affinity nerve growth factor receptor	NTRK1	11
55	P22894	Neutrophil collagenase	MMP8	11
56	Q99814	Endothelial PAS domain-containing protein 1	EPAS1	10
57	P10415	Apoptosis regulator Bcl-2	BCL2	10
58	P32246	C-C chemokine receptor type 1	CCR1	8
59	P11413	Glucose-6-phosphate 1-dehydrogenase	G6PD	8
60	P08123	Collagen alpha-2	COL1A2	8
61	P23219	Prostaglandin G/H synthase 1	PTGS1	8
62	Q13133	Oxysterols receptor LXR-alpha	NR1H3	8
63	O75116	Rho-associated protein kinase 2	ROCK2	8
64	Q03181	Peroxisome proliferator-activated receptor delta	PPARD	7
65	P25105	Platelet-activating factor receptor	PTAFR	7
66	P27169	Serum paraoxonase/arylesterase 1	PON1	7
67	P17706	Tyrosine-protein phosphatase nonreceptor type 2	PTPN2	7
68	O75469	Nuclear receptor subfamily 1 group I member 2	NR1I2	6
69	P28223	5-Hydroxytryptamine receptor 2A	HTR2A	6
70	P12104	Fatty acid-binding protein, intestinal	FABP2	6
71	P02753	Retinol-binding protein 4	RBP4	5
72	P39900	Macrophage metalloelastase	MMP12	5
73	P08235	Mineralocorticoid receptor	NR3C2	5
74	P11597	Cholesteryl ester transfer protein	CETP	5
75	P07477	Trypsin-1	PRSS1	4
76	Q07973	1,25-Dihydroxyvitamin D	CYP24A1	4
77	P30542	Adenosine receptor A1	ADORA1	4
78	P18054	Polyunsaturated fatty acid lipoxygenase ALOX12	ALOX12	4
79	O00763	Acetyl-CoA carboxylase 2	ACACB	4
80	P04278	Sex hormone-binding globulin	SHBG	3
81	P80365	Corticosteroid 11-beta-dehydrogenase isozyme 2	HSD11B2	3
82	P13866	Sodium/glucose cotransporter 1	SLC5A1	2
83	P35610	Sterol O-acyltransferase 1	SOAT1	1
84	P05091	Aldehyde dehydrogenase, mitochondrial	ALDH2	1

## Data Availability

The data used to support the findings of this study are included within the article.
